# Surgical Treatment Strategy of Functional Tricuspid Regurgitation

**DOI:** 10.31083/j.rcm2505182

**Published:** 2024-05-21

**Authors:** Peihe Wang, Yu Huang, Lu Sun, Zhen Han

**Affiliations:** ^1^Department of Cardiovascular Surgery, Peking University Shenzhen Hospital, 518000 Shenzhen, Guangdong, China; ^2^Shenzhen Clinical Institute of Shantou University Medical College, 518000 Shenzhen, Guangdong, China

**Keywords:** tricuspid valve, functional tricuspid regurgitation, tricuspid regurgitation, tricuspid valve regurgitation, surgery, tricuspid valve repair, tricuspid valve replacement

## Abstract

Functional tricuspid regurgitation (FTR) is a common type of tricuspid regurgitation (TR), particularly in cases of left heart valve disease. Historically, cardiac surgeons have not placed much emphasis on FTR and instead focused primarily on managing left heart valve disease. However, as research has progressed, it has become evident that severe TR significantly impacts the prognosis of heart valve surgery. Furthermore, significant improvements in postoperative cardiac function and quality of life have been observed when addressing the tricuspid valve alongside left heart disease management. This article aims to review current approaches for and timing of the surgical management of FTR while also analyzing the limitations of existing tricuspid surgical strategies.

## 1. Introduction

Historically, the tricuspid valve has usually been overlooked because it did not play an extraordinarily important function role like the mitral valve and aortic valve and was considered a “dispensable” heart valve. However, with the aging of the population and the increasing annual incidence of cardiovascular disease, tricuspid valve disease has attracted increasing attention from cardiac surgeons, and more research on treatment measures for the tricuspid valve has been carried out [1, 2]. At the same time, we must be concerned that with the increase in the number of patients using cardiac implantable electronic devices (cardioverter-defibrillators or pacemakers, etc.), the incidence of TR is increased by the resultant medically-induced injuries, making the diagnosis and management of tricuspid regurgitation a matter that needs to be taken more seriously [3, 4, 5].

Tricuspid regurgitation (TR) is the most common manifestation of tricuspid valve disease, with functional tricuspid regurgitation (FTR) accounting for the vast majority of cases [6], ranging from approximately 70–90% of TR cases [7, 8]. FTR is often caused by valvular disease resulting from left-sided heart issues and has been observed in a significant percentage of patients with mitral valve disease, ranging from 26% to 53% [9, 10]. Recent studies have found that 86% of tricuspid valve procedures are performed at the same time as left-sided heart valve surgery, with the most common combined procedure being mitral valve surgery, which accounts for approximately 79% of all cases, and tricuspid valve surgery performed at the same time as aortic valve surgery, which is performed in approximately 10% of patients [11, 12, 13]. The emergence of minimally invasive vascular surgery has led to the rise of percutaneous tricuspid valve surgery as a viable option for treating TR.

However, due to the shorter lifespan of patients with FTR and timing limitations, FTR is still commonly treated simultaneously with left-sided heart valve surgery [14]. Despite its effectiveness, surgical treatments for FTR have limitations, including the possibility of recurrence in the medium to long term after surgery [15]. As a result, cardiovascular surgeons have made significant efforts to innovate and improve treatments for TR to achieve longer-term benefits. This article reviews modalities for the surgical treatment of the tricuspid valve and analyzes the shortcomings of these modalities in an effort to provide cardiovascular surgeons with a better understanding of the tricuspid valve.

## 2. Tricuspid Valve Structure

The tricuspid valve is the largest, rightmost and topmost of the heart valves, consisting of the annulus, leaflets and subvalvular structures (papillary muscles, spinal cord attachments) [16]. The leaflets and subvalvular structures circulate during contraction and relaxation of the right ventricle in a “closed-open” process, thus serving to keep the blood flowing in one direction. The tricuspid valve typically measures 7–9 ${\mathrm{cm}}^{2}$ in size and the right ventricle contracts less forcefully than the left ventricle [17]. This results in a smaller pressure difference between the ventricular and atrial sides of the tricuspid valve and slower blood flow through it. Due to the need for postoperative anticoagulation therapy, replacement of the tricuspid valve is rarely considered a treatment option for tricuspid valve disease. The tricuspid valve generally consists of 3 leaflets, which are usually referred to as the septal leaflet, anterior leaflet, and posterior leaflet [18]. The anterior leaflet is usually considered to be the largest and longest in the radial direction, with the largest area and the largest amplitude of motion. The leaflets of the tricuspid valve fuse at the level of or slightly below the annulus, with a fusion length of approximately 5–10 mm, which provides the potential for deformation of the tricuspid valve in the event of a lesion. Hahn *et al*. [19] innovatively proposed a new class scheme for tricuspid valve (TV) leaflet nomenclature by retrospectively analyzing patients from four medical centers: four morphological types were identified: type I, 3 leaflets; type II, 2 leaflets; type IIIA, 4 leaflets with 2 anterior; type IIIB, 4 leaflets with 2 posterior; type IIIC, 4 leaflets with 2 septal; and type IV, >4 leaflets. This classification scheme provides new understanding of the surgical management of the tricuspid valve.

The normal tricuspid annulus is horseshoe shaped and was previously superficially thought to be planar, but on 3 dimensional (3D) echocardiography and magnetic resonance imaging, etc., it was found not to be planar [20]. Unlike the mitral annulus, the tricuspid annulus is a dynamic structure and may be difficult to identify during surgical or anatomic examination. The papillary muscle and notochord form the subvalvular structure of the tricuspid valve. It is generally believed that the tricuspid valve contains 3 papillary muscles, including the anterior papillary muscle and its notochord attached to the anterior and posterior leaflets, and the posterior papillary muscle and its notochord attached to the posterior and septal leaflets [21].

The location of the septal papillary muscle is flexible and variable, but not everyone has a septal papillary muscle. The location of papillary muscle attachment is also regular. The posterior and septal papillary muscles generally attach to the interventricular septum, and the anterior papillary muscle attaches to the anterolateral wall of the right ventricle; thus when the spatial structure of the right ventricle changes, the spatial relationships among of the tricuspid valve leaflets and the attached papillary muscles will also change.

## 3. Pathophysiological Process

TR is classified as primary or secondary, depending on the etiology. Secondary TR, also often referred to as FTR, is the most common type of TR and is, therefore, the focus of attention. In particular, TR due to left heart disease accounts for a large proportion of FTR cases [22, 23]. The pathogenesis of FTR is multifactorial and complex. FTR can be classified according to the site of primary disease into the following categories: (1) FTR due to left heart disease; (2) FTR due to pulmonary hypertension; (3) FTR due to right ventricular dysfunction; and (4) FTR of undetected cause (idiopathic FTR) [24]. The recent division of TR into atrial TR (the primary mechanism is atrial dilation, usually in the setting of atrial fibrillation) and ventricular TR (the primary mechanism is right ventricular dilation in the setting of biventricular heart failure or pulmonary hypertension) has provided new ideas for the study of TR [25].

The most common causes of FTR are left heart valve disease (mainly mitral valve disease), left and right ventricular cardiomyopathy (ischemic and nonischemic), and right ventricular dilatation due to pulmonary disease (pulmonary heart disease) [23]. In left-sided heart valve disease, such as mitral stenosis or insufficiency, increased mitral valve preload and afterload leads to increased pressure in the left atrium and consequent left heart failure which in turn causes the transmission of pressure through the pulmonary veins to the pulmonary arteries, resulting in pulmonary hypertension and impaired contraction of the right ventricle. Over time, intraventricular pressure increases in the right ventricle and the ventricular cavity enlarges, resulting in dilatation of the tricuspid annulus or a change in the spatial positions of the papillary muscles [26]. This creates a chain of disease transmission consisting of the left ventricle, left atrium, pulmonary artery, right ventricle, and tricuspid valve. Disease occurring anywhere in this chain may, in time, cause a change in the spatial structure of the tricuspid valve, in turn leading to the development of TR.

## 4. Left Heart Valve Surgery Performed at the Same Time as Tricuspid Valve Surgery

FTR has not previously been treated surgically, but only conservatively, and it was thought that TR would improve significantly after correction of left heart valve disease [27]. With the development of surgical treatment modalities and postoperative follow-up studies, it has been observed that TR does not resolve as well as expected in patients with left heart valve disease combined with TR [28]. Additionally, postoperative follow-up has revealed that severe TR is strongly associated with early and mid-to-late mortality in patients after left heart valve surgery [29, 30, 31]. This is why it is particularly important to perform left heart valve surgery with simultaneous tricuspid valve repair [32].

Tricuspid valve morphologic evaluation is mostly accomplished by transthoracic and transesophageal echocardiography (3-dimensional echocardiography), which is essential for differentiating the etiology and mechanism of TR and for selecting appropriate structural interventions or surgery. Transesophageal echocardiography is considered the imaging modality of choice for comprehensive intraoperative assessment of tricuspid valve status and for guiding surgery. Clinical grading of TR is based on the regurgitant jet area on color Doppler: mild (jet area <1.0 ${\mathrm{cm}}^{2}$), mild (jet area 1–5 ${\mathrm{cm}}^{2}$), moderate (jet area 5–10 ${\mathrm{cm}}^{2}$), and severe (jet area >10 ${\mathrm{cm}}^{2}$) [33, 34]. The use of this metric to assess the severity of TR is problematic; because the spatial structure of the tricuspid annulus is not planar or fixed under complex hemodynamics, and the range of regurgitant jet area on color Doppler does not reflect the regurgitant volume. Thus, studies have been performed to propose a more reasonable evaluation [29, 35]. 3D vena contracta (VC) area has better correlation with effective regurgitant orifice area (EROA) compared to 2 dimensional (2D) vena contracta width, so 3D ultrasound helps to better depict regurgitant orifice morphology and grade TR severity. Recently, some scholars have called for expanding the grading of TR based on recent findings, and severe TR can be subdivided into severe, massive, and stormy flow [36, 37, 38]. The latest and widely used echocardiographic classification methods for tricuspid regurgitation are shown in Table 1.

**Table 1.** **Grading the severity of tricuspid regurgitation**.

TR Severity classes	Mild	Moderate	Severe	Massive	Torrential
VC width (mm)	<3	3–6.9	7–13	14–20	≥21
3D VC (${\mathrm{mm}}^{2}$)			75–94	95–114	≥115
R Vol (mL)	<30	30–44	45–59	60–74	≥75
RF (%)	<30	30–49	≥50		
EROA (${\mathrm{mm}}^{2}$)	<20	20–39	40–59	60–79	≥80

TR, tricuspid regurgitation; VC, vena contracta; R Vol, regurgitant volume; RF, regurgitant fraction; EROA, effective regurgitant orifice area; 3D, 3 dimensional.

The timing of surgery is critical. Appropriate timing of intervention on the tricuspid valve can reduce or avoid right heart insufficiency and corresponding postoperative complications [11, 39]. Treating TR at the right time can slow the progression of the disease while improving patient survival and quality of life.

**Fig. 1.**
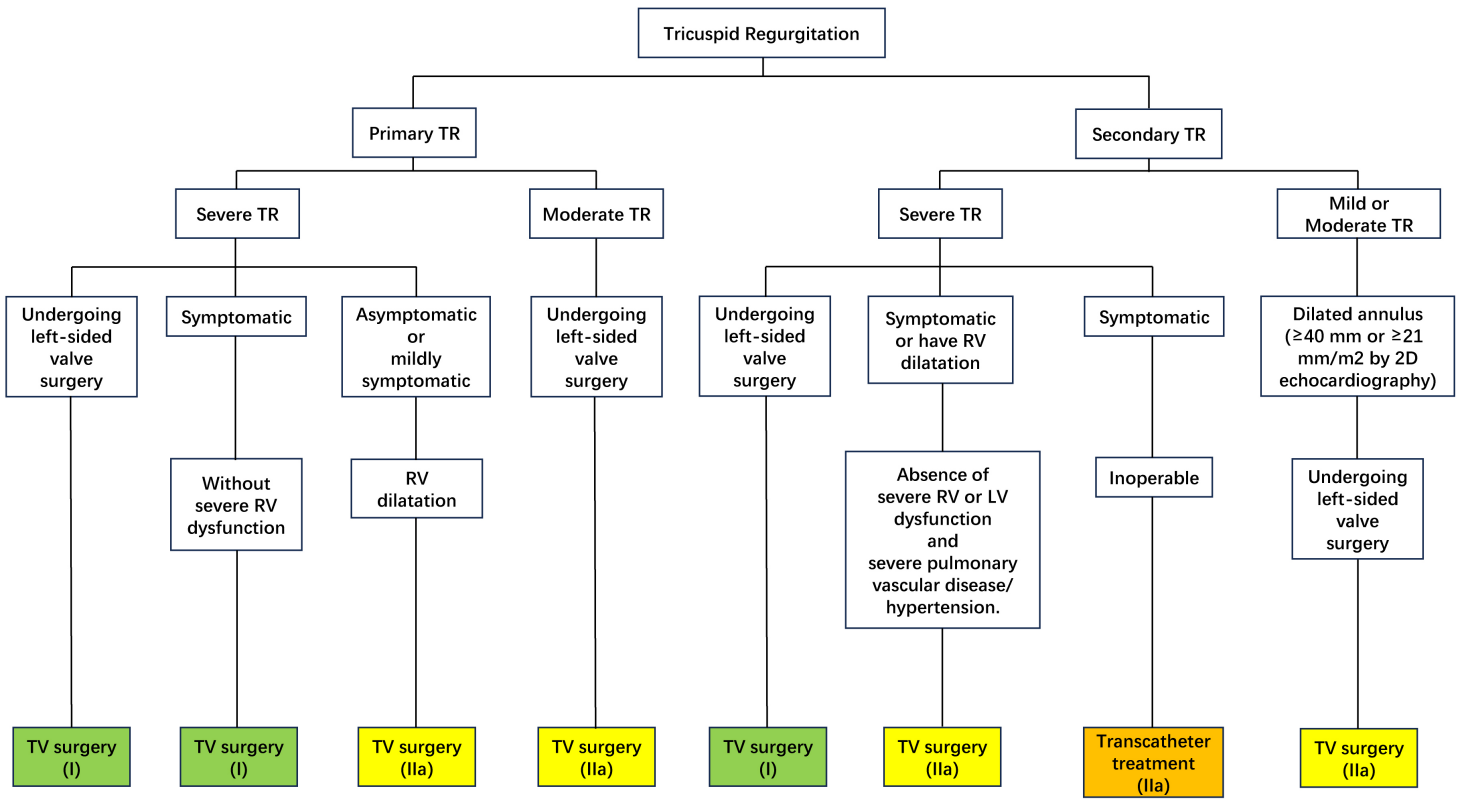
**Recommendations on the timing of surgery for tricuspid regurgitation**. TR, tricuspid regurgitation; TV, tricuspid valve; RV, right ventricle; LV, left ventricle; 2D, 2 dimensional.

The 2020 American College of Cardiology/American Heart Association (ACC/AHA) Guidelines for the Management of Patients with Valvular Heart Disease provide a Class of Recommendation 1 Level for patients with severe TR (Stages C and D) undergoing left-sided valve surgery and recommend tricuspid valve surgery [40, 41, 42]. The 2021 European Society of Cardiology/European Association for Cardio-Thoracic Surgery (ESC/EACTS) Guidelines for the Management of Heart Valve Disease similarly recommend surgery for patients with severe tricuspid stenosis undergoing left-sided valve intervention [43]. They also simultaneously recommended that in patients with progressive TR (stage B) undergoing left-sided valve surgery, simultaneous tricuspid valve surgery is also beneficial in the context of tricuspid annular dilatation (tricuspid annular end-diastolic diameter >4.0 cm or >21 mm/$m^{2}$ by 2D echocardiography) or of previous signs and symptoms of right-sided heart failure, even in the absence of severe TR [40, 44]. In the absence of severe right or left ventricular dysfunction and severe pulmonary vascular disease/hypertension, when patients with severe secondary TR are symptomatic or have right ventricular dilatation, it is recommended that surgical treatment of the tricuspid valve should be performed concomitantly with surgical treatment of the left heart valve [43, 45, 46]. Fig. 1 lists the recommendations on the timing of surgery for tricuspid regurgitation.

There is no uniform conclusion regarding whether patients with left heart valve disease without right heart symptoms or imaging manifestations of tricuspid valve malfunction also require pretreatment of the tricuspid valve. According to recent research, cardiac surgery in patients with simple left heart valve disease without significant TR is still followed by a significant increase in the degree of TR in some patients compared with the degree of TR before surgery [47, 48, 49, 50, 51].

## 5. Types of Surgery for TR

At one time there was considerable controversy over whether tricuspid valves should be replaced or repaired, and it was found that tricuspid valve repair provided a better prognosis [27]. To effectively address TR and improve the survival and quality of life of patients after cardiac surgery, various tricuspid valve surgical procedures have been invented, such as those invented by Bex [52], Minale [53], Kay [54], and Reed [55], which have been abandoned due to poor results. De Vega annuloplasty and ring annuloplasty are widely used in clinical practice, but as research has progressed, a large number of randomized controlled studies have found ring annuloplasty to have more significant advantages in terms of the degree of improvement in TR and the effective duration of the procedure [56, 57]. In the following section, we discuss several of the more widely used tricuspid valve surgical procedures in clinical practice today. The results of the various surgical modalities are presented in tabular form in **Supplementary Table 1**.

### 5.1 De Vega Annuloplasty

In 1972, Dr. De Vega pioneered the surgical treatment of the tricuspid valve by proposing an original, selective, adjustable permanent tricuspid valvuloplasty [58]. In honor of the great doctor’s contributions, the procedure was named De Vega annuloplasty. This procedure is favored by a wide range of cardiac surgeons for its ability to achieve satisfactory surgical results through a simple, cost-effective method.

**Fig. 2.**
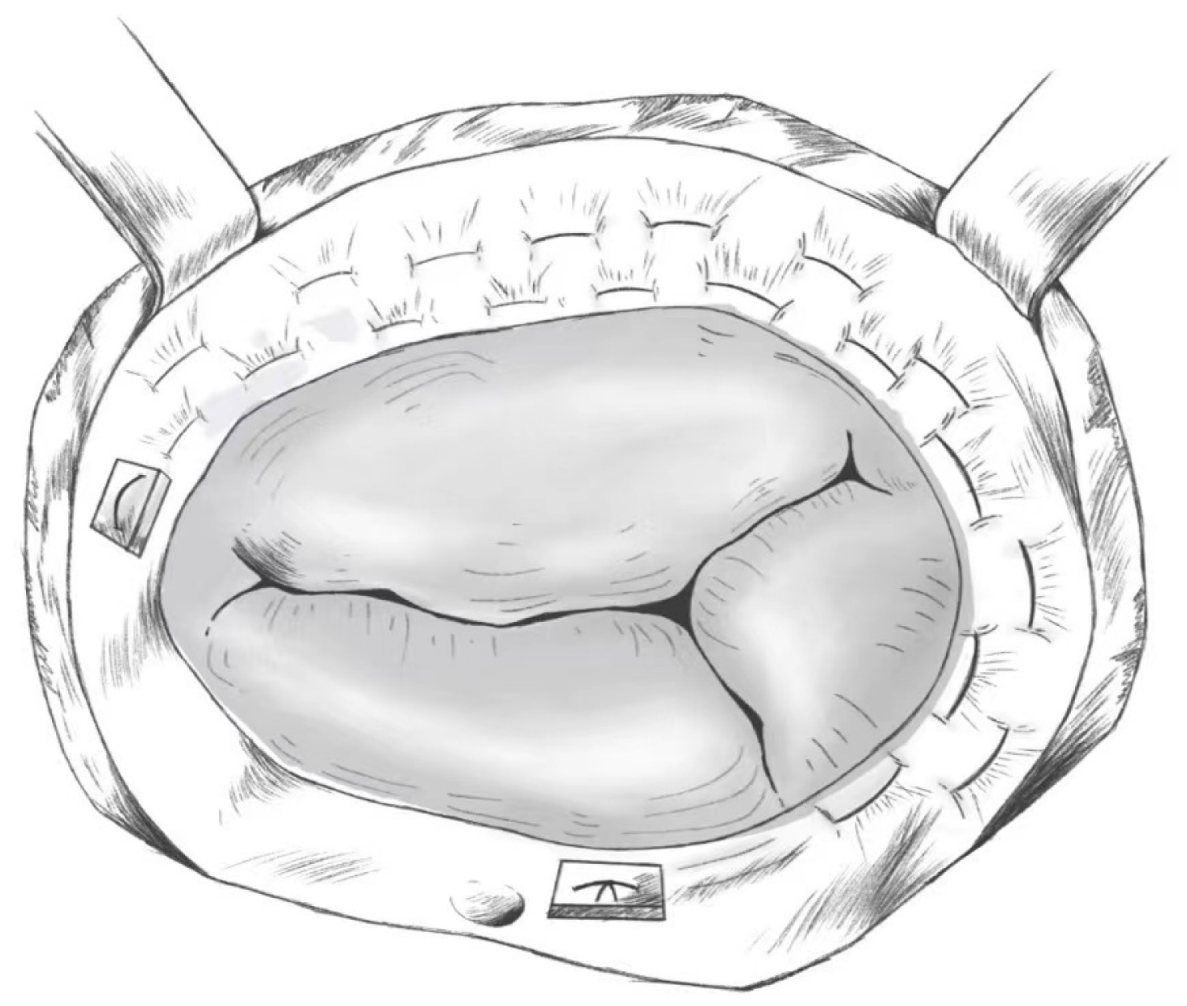
**De Vega Annuloplasty**.

This procedure is performed by using a 2-0 or 3-0 double-needle polyester suture with a Teflon spacer to perform a continuous purse-string suture from the posterior septal junction to the anterior septal junction, with the point of entry at the annulus or right ventricular free wall junction, taking care to avoid the septal annulus. The second stitch is placed parallel to the first stitch, 1–3 mm higher, with both sutures alternating clockwise relative to the first stitch. The depth of the needle is approximately 2 mm and 10–12 stitches are made in each suture [59]. Two needles are threaded through the other spacer at the junction of the front compartment. The two sutures are tightened after the suturing is completed thereby creating a purse-string effect and narrowing the entire annular orifice; 2–3 fingers can be placed more snugly into the optimally contracted tricuspid orifice, or measurement tools such as an annuloplasty gauge can be used instead. Finally, a water injection test is performed to detect flap closure [60]. A model diagram is shown in Fig. 2.

De Vega annuloplasty is a form of early tricuspid valve repair that was widely used in early clinical practice with satisfactory results [61, 62]. As clinical practice has progressed, more research has been done on this repair, and the disadvantages of this repair have been increasingly recognized. Re-regurgitation of more than moderate class was observed in a higher number of patients in the mid- to long-term postoperative period [47, 63, 64]. Due to the special spatial structure of the tricuspid valve, De Vega annuloplasty has significant shortcomings in terms of the durability of the contouring of the tricuspid annulus [65].

### 5.2 Ring Annuloplasty

**Fig. 3.**
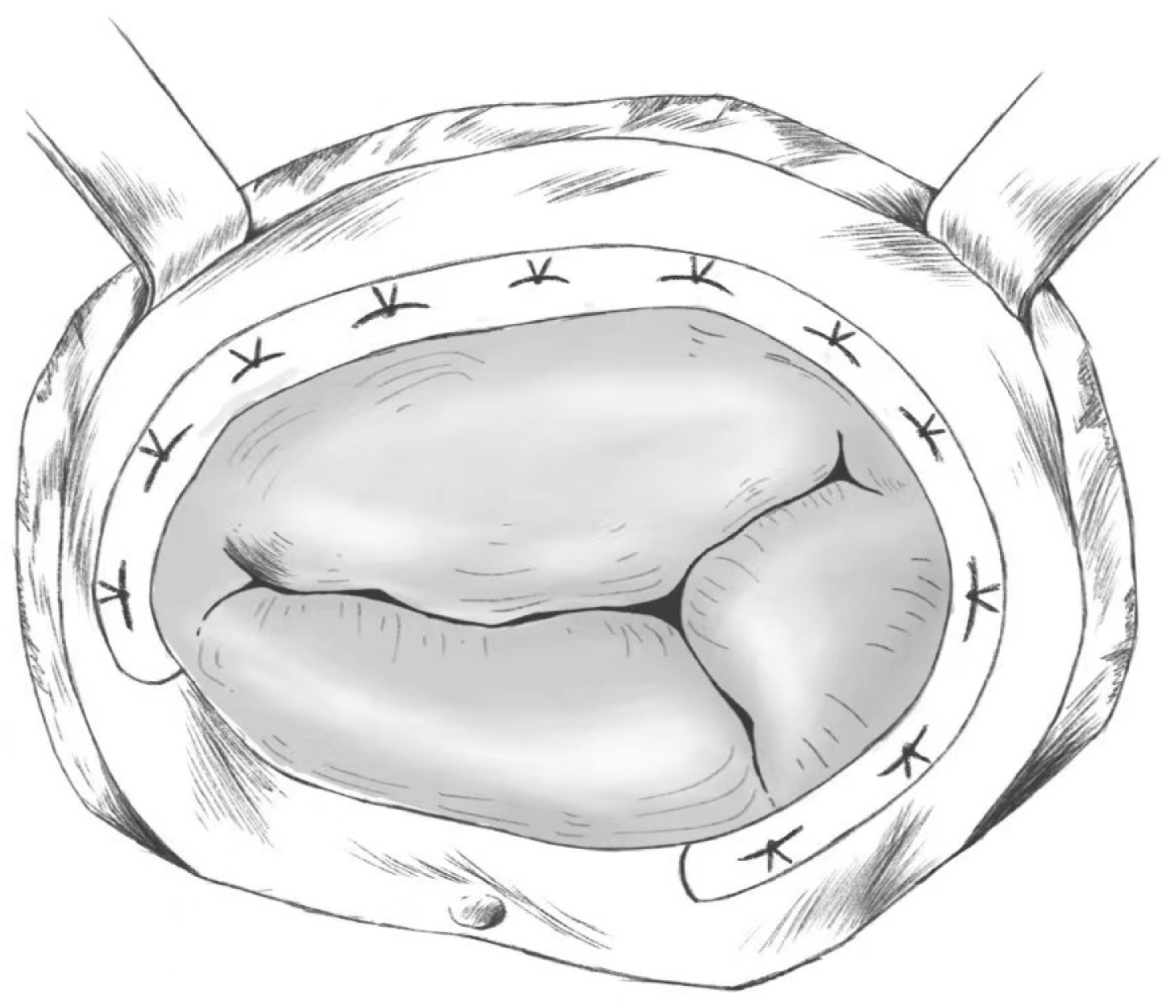
**Ring Annuloplasty**.

The ring annuloplasty approach involves horizontal mattress suturing along the tricuspid valve with multiple 3-0 TiCeron sutures, with a wider stitch spacing on the annulus and a smaller stitch spacing on the pedunculated ring, which serves to narrow the annulus; additionally, gentle, transverse pinching and pulling of the leaflets help determine the point of attachment of the leaflets, as well as the security of the annulus and subvalvular structures [60]. A certain depth of entry is also important to avoid tearing the annulus when tightening the sutures, and it is important to take care to avoid adjoining structures and not damage the aortic root, the right coronary artery, or the atrioventricular conduction system. This procedure can be considered a modified version of the poor ring contouring in De Vega procedure. It improves on the De Vega annuloplasty by allowing the tricuspid annulus to maintain a more stable shape while providing better rigidity such that the results last longer [66]. A model diagram is shown in Fig. 3.

Ring Annuloplasty has a significant advantage over De Vega Annuloplasty in improving the degree of tricuspid regurgitation and has a lower re-regurgitation rate [57, 67, 68, 69]. However, some problems have been identified in the clinic, such as repair failures due to broken molding rings [70, 71]. The effectiveness and success of Ring Annuloplasty continues to be improved with the use of new molded rings [72].

### 5.3 Defoliation Technology

**Fig. 4.**
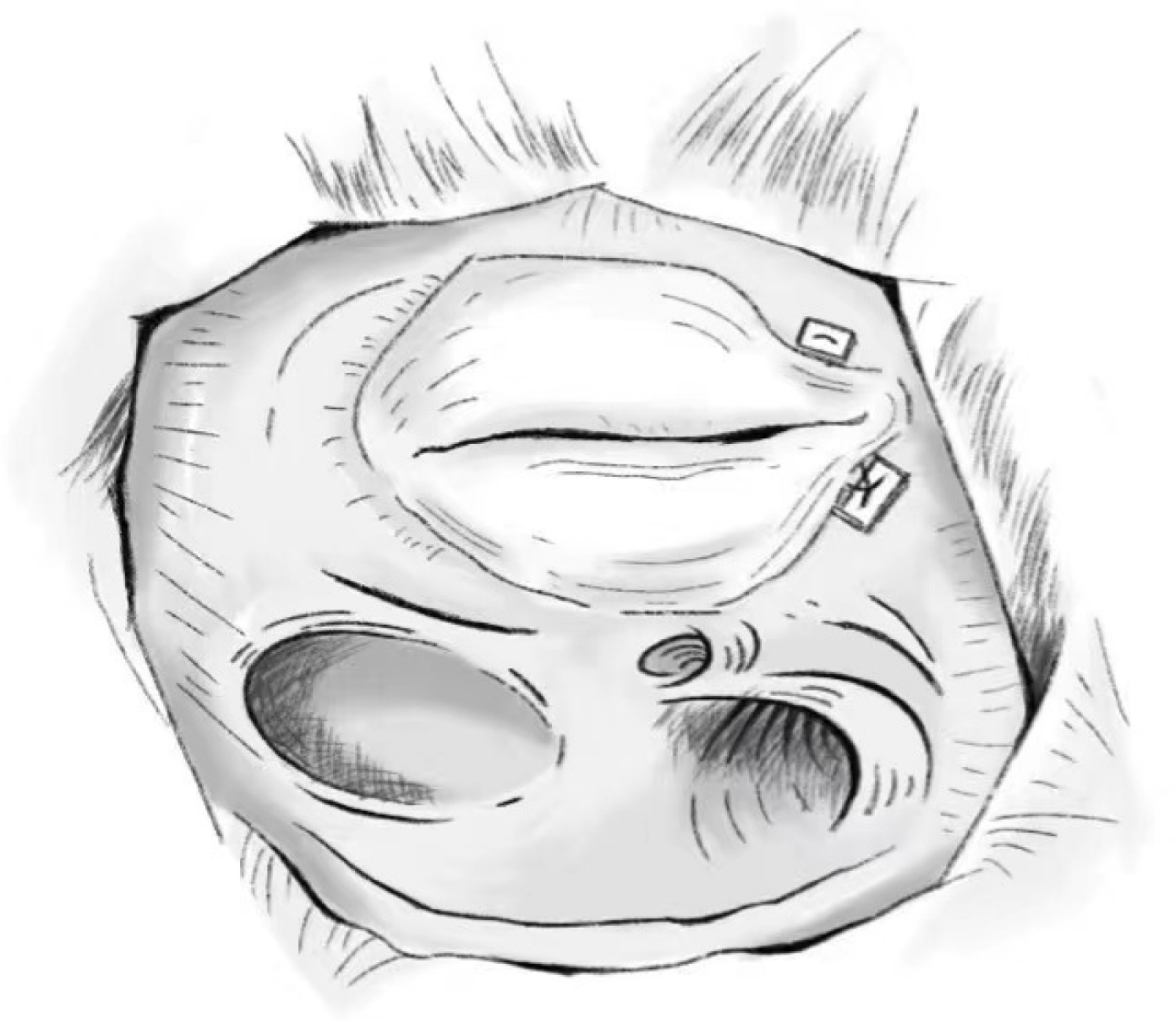
**Difoliation Technology**.

The posterior valve annulus is folded to create a structure in which the posterior valve is removed, at which point the tricuspid valve is changed from three leaflets to two leaflets, achieving a reduction in the orifice. A 2-0 spacer-bearing polypropylene suture is used; the needle enters through the flap ring at the junction of the anterior and posterior flaps, exits from the posterior flap, and passes through the flap ring at the junction of the posterior septum. This maneuver is not in the atrioventricular node area, but extra care should still be taken so that the suture needle does not cross the coronary sinus orifice. The diameter of the repaired tricuspid valve should return to normal [60]. A model diagram is shown in Fig. 4. Due to the difficulty of this type of repair and the presence of damaging sutures to the tricuspid annulus, this type of repair is not widely used in clinical practice.

### 5.4 Subvalvular Surgical Techniques

In addition to surgery on the flap annulus, studies have also focused on subvalvular structures and have obtained better results in animal and clinically studies [73, 74, 75]. Recently, Takeshita *et al*. [76] proposed a new subvalvular surgical technique based on ring annuloplasty, which consists of annular repositioning, papillary muscle repositioning, and papillary muscle strapping. The first step is to perform ring annuloplasty. A 4-0 polytetrafluoroethylene double-needle suture with spacers and gauze is sutured to the septum near the base of the papillary muscle of the interventricular septum, and the suture is then threaded through the posterior septal portion of the flap ring near the coronary sinus orifice and the corresponding flap annuloplasty ring, where the flap annuloplasty ring is then implanted. Afterward, another 4-0 polytetrafluoroethylene double-needle suture with spacers is used to suture the base of the anterior papillary muscle, with the free end passing through the corresponding anterior annulus and then through the ring on the tricuspid valve annulus, ending in that ring. This step requires a water-fill test to determine the appropriate length of the suture. The CV-4 suture is tied as the anterior leaflet approaches the plane of the tricuspid annulus. The final step is to perform papillary muscle strapping; this step then does not require ring annuloplasty first. All papillary muscles between the base of the papillary muscles and the free wall of the right ventricle are closed using CV-0 sutures starting from the around base of the anterior papillary muscle. The suture is threaded through the papillary muscle gap near the ventricular septum and tightened. This allows the anterior and posterior leaflets to be brought close to the level of the annular plane to restore tricuspid valve closure in the presence of abnormal tendon cords or papillary muscles.

Subvalvular Surgical Techniques have advantages in tricuspid regurgitation caused by subvalvular lesions and other factors, enabling individualized treatment of such patients.

### 5.5 Valve Replacement

**Fig. 5.**
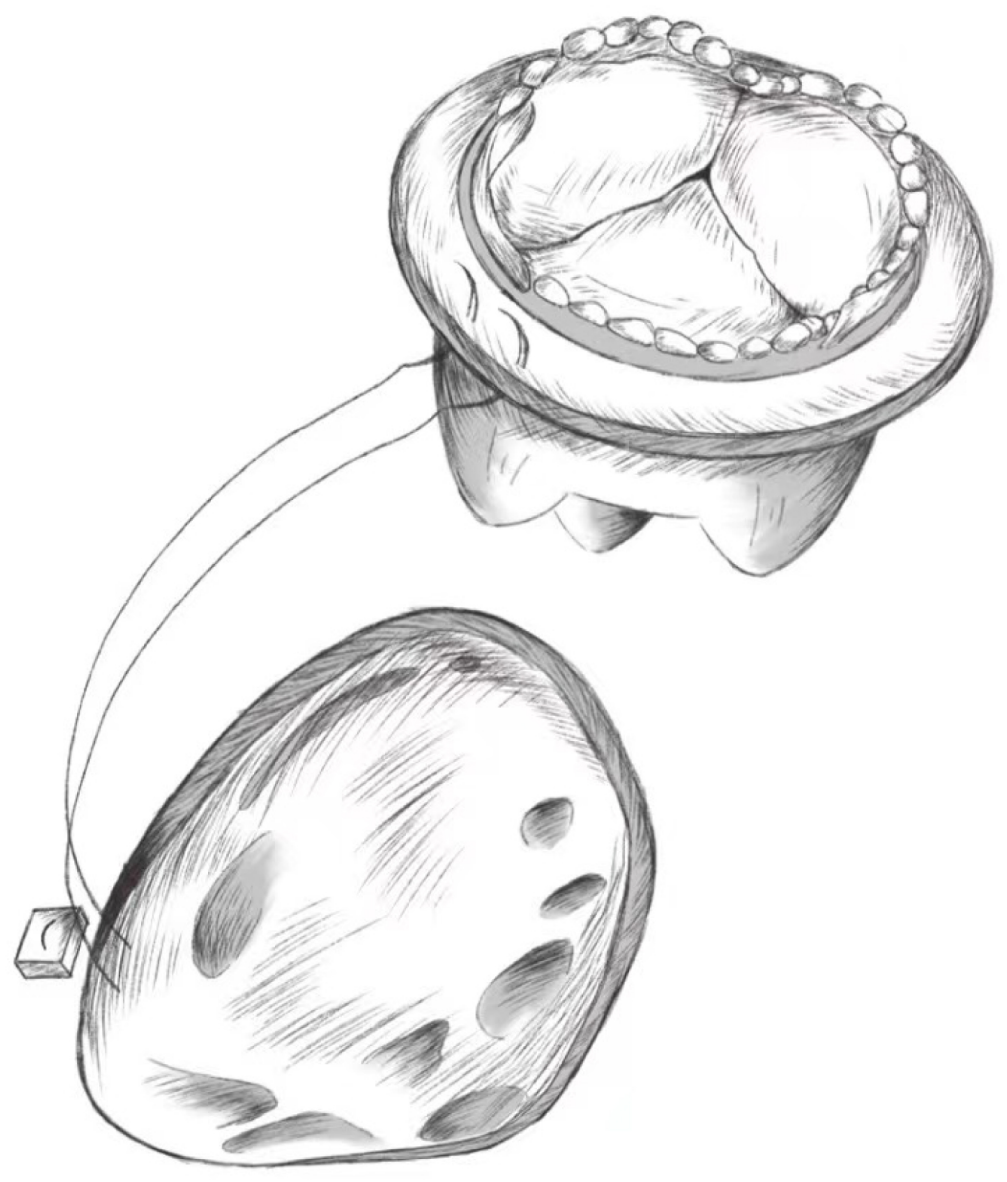
**Tricuspid Valve Replacement**.

An important principle of tricuspid valve surgery is to avoid valve replacement if valve repair is possible [77]. Mortality after tricuspid valve replacement is significantly higher than after tricuspid valve repair [78, 79, 80]. However, if the tricuspid valve is severely diseased or a satisfactory clinical outcome cannot be achieved after repair, tricuspid valve replacement becomes an option that that has to be considered [40, 80, 81]. When performing tricuspid valve replacement, an appropriate prosthetic valve is usually selected based on the size of the annulus, and as much subvalvular tissue as possible is preserved. In certain patients with infective endocarditis, care is taken to preserve 2–3 mm of leaflet tissue while completely removing the tricuspid valve leaflets and subvalvular tissue to avoid damage to the surrounding conduction system. The position of the placed tricuspid valve should also be checked to avoid contact between the prosthetic valve and the preserved subvalvular tissue that could interfere with movement of the prosthetic valve leaflets. A model diagram is shown in Fig. 5.

Using a 2-0 double-needle polyester suture with a Teflon spacer through the tricuspid valve annulus, the suture is passed over the suture ring of the prosthetic valve that has been placed in the expected position, with being tightened. A suitable attachment area is chosen to avoid damage to the conduction system, and the above suture steps are repeated 3–5 mm away from the previous suture position. After the prosthetic annulus is completely sutured to the tricuspid annulus, the sutures are tightened, the prosthetic valve is slowly pushed into the tricuspid orifice; each suture is knotted separately after checking for accuracy. Tying the knots ensures that the prosthetic valve fits tightly against the tricuspid annulus to avoid perivalvular leakage in the postoperative period.

In patients with tricuspid regurgitation, whether repair or replacement of the tricuspid valve is more beneficial to the patient is a matter of curiosity for surgeons. In a study by Rawan Alghamdi *et al*. [82] in a 5-year prognostic propensity score-matched analysis of tricuspid valve repair versus replacement, they found that the 30-day mortality rate was 3.79% in the valve repair group versus 1.89% in the valve replacement group, but after adjusted for this, there was no significant difference between the two groups. Sameh M. Said *et al*. [83] studied tricuspid regurgitation in congenital heart disease and after following patients for 4.5 years found that tricuspid valve repair had a low early mortality rate and a high late survival rate compared to valve replacement. Overall early mortality was 3.1%, 2.5% in the valve repair group and 5.4% in the valve replacement group (*p* < 0.001) [83]. The high mortality rate of valve replacement may be due to selection bias, as valve replacement is chosen for patients with more advanced end-stage annular dilatation and in patients with poor ventricular function to avoid reoperation. Ying Huang *et al*. [4] conducted a study to evaluate the outcome of surgical treatment of lead-induced tricuspid regurgitation in patients with congenital heart disease and found that the overall 5-year survival rate was 80.4%, with 5-year survival rates of 79.7% and 81.4% in the repair and replacement groups, respectively, and comparable survival and television reintervention rates in both groups. TR improved in both groups after surgery (both *p* < 0.001), but was better in the replacement group than in the repair group (*p* < 0.001) [4]. Some studies have reached various conclusions regarding the difference between valve repair and valve replacement. Although there is may no significant difference between valve repair and valve replacement in terms of mortality, valve replacement has a relative advantage in terms of improvement in tricuspid regurgitation. We believe that patients with severe functional tricuspid regurgitation should be offered early valve repair when possible.

The choice of mechanical or biologic valves is a topic that is never more relevant in valve replacement. Chad J. Zack *et al*. [84] found in a study that the mortality rate was 5.9% for patients with TV valve repair, 9.1% for patients with bioprosthetic valve replacement, and 13.6% for patients with mechanical valve replacement (*p* < 0.003). A Meta-analysis by Sherif Negm *et al*. [85] on the difference between the use of mechanical and biologic valves for tricuspid valve replacement showed that patients with mechanical tricuspid valve replacement and biologic tricuspid valve replacement were at equal risk for 30-day and late death, reoperation, and 5-year valve failure. According to current research opinion, there is no significant difference in mortality between tricuspid valve replacement using mechanical and bioprosthetic valves.

Bioprosthetic valves do not require lifelong antithrombotic therapy, only a period of oral anticoagulation after surgery [43]. At the same time, bioprosthetic valves allow secondary pacemaker implantation in the right ventricle and right heart catheterization, preserving the possibility of transcatheter reoperation [86, 87, 88]. However, the durability of the bioprosthetic valve is another issue to consider. In a large single-institution survey, the freedom from 15-year degradation of bioprosthetic valves was 83% [89]. Similarly, in another retrospective clinical study with a follow-up time of up to 15 years, long-term survival and tricuspid-related events for bioprosthetic TVR were found to be comparable to the results for mechanical TVR [90]. The effectiveness of the bioprosthetic valve at 10 and 15 years was 91.2% and 78.8%, respectively. As technology has evolved, the lifespan of biologic valves has continued to increase, and reoperation rates have been significantly reduced, although there are still some disparities compared to mechanical valves. Compared to biologic valves, mechanical valves have a much longer lifespan, which is a very important advantage. However, mechanical valves also have their own fatal disadvantage — postoperative anticoagulation — which prevents doctors and patients from prioritizing them when choosing a valve type. As current statistics show, only 10–15% of patients with tricuspid regurgitation undergo valve replacement, and approximately 85–90% of these patients who undergo valve replacement choose a bioprosthetic valve [91]. Equally interesting findings were that more than 90% of patients undergoing combined valve surgery opted for valve repair when dealing with the tricuspid valve, whereas about 60 of patients with isolated tricuspid regurgitation underwent valve replacement [92, 93].

The selection of the type of tricuspid prosthetic valve, follows the same principles used for the selection of other prosthetic valves and needs to take into account the patient’s age, the use of anticoagulant drugs, and the patient’s wishes. It is generally accepted that mechanical flaps can be used in younger patients without contraindications to anticoagulation therapy [40]. In tricuspid valve replacement, the generally accepted opinion is that treatment with a bioprosthetic valve does not require postoperative anticoagulation therapy [94, 95]. In conclusion, when considering tricuspid valve replacement, it is necessary to take into account the patient’s age, willingness to use anticoagulants, clinical status, type of disease, and social factors to develop a personalized and specialized treatment plan for the patient.

## 6. Current Problems in Surgical Treatment

The introduction of tricuspid valve surgery has improved the prognosis and quality of life of a large number of patients with FTR, but an increasing number of shortcomings have been identified over time. Although De Vega annuloplasty is effective in reducing the orifice area to relieve TR, due to poor valve plasticity with respect to the orifice, a proportion of patients show TR recurrence after prolonged postoperative follow-up, with more severe regurgitation [47, 65, 67]. Approximately 12% of patients who undergo De Vega annuloplasty require reoperation, which is significantly higher than the 2% reoperation rate for ring annuloplasty [96]. Although ring annuloplasty is superior to De Vega annuloplasty in reducing TR and in reliability, it still has shortcomings. Leaflet embolism has been found to be an important factor in residual regurgitation after tricuspid annuloplasty; thus ring annuloplasty is not effective in improving FTR caused by pressure overload [97]. It has also been found that TR was not completely eliminated after annuloplasty, with leaving persistent TR in all cases after repair [57]. Transcatheter tricuspid valve therapy is another option to consider for some patients with inoperable TR or FTR who have undergone tricuspid valve surgery but are not candidates for reoperation [98].

Severe tricuspid regurgitation can likewise occur in various congenital heart diseases. Tricuspid regurgitation due to congenital heart disease can also be treated with valve repair and valve replacement. The outcome of valve replacement or valve repair was similar to that of patients with functional tricuspid regurgitation and isolated tricuspid regurgitation. It can improve tricuspid regurgitation well and has a low mortality and recurrence rate of tricuspid regurgitation [83]. A single-center retrospective study of patients with congenital heart disease who underwent televised surgery found that annuloplasty had a lower recurrence rate of moderate to severe regurgitation compared with sutureplasty repair [99]. Congenital heart disease is not the focus of this article, but tricuspid valve disease due to congenital heart disease still requires further attention.

In addition to left heart valve lesion-associated FTR, isolated FTR has received equal attention from many scholars. Atrial fibrillation-associated TR and TR occurring late after left-sided valve surgery are currently considered the main causes of isolated FTR [100]. The main pathophysiological processes are increased right ventricular preload and afterload and atrial arrhythmias leading to anatomical and functional right heart chamber remodelling interacting with the tricuspid valve apparatus, which in turn leads to tricuspid valve insufficiency [101, 102]. Right heart function is considered a key factor in determining the timing of surgical treatment of such tricuspid valve disease. There is still some controversy about the timing and strategy of surgery for these high-risk patients. Jinmiao Chen *et al*. [103] concluded that surgical intervention is necessary before patients develop known risk factors such as anaemia and liver dysfunction. Several studies have concluded that patients with severe TR should be referred early in the course of the disease to avoid TV interventions in the late and desperate stages of the disease [93, 104].

With the development of new approaches, patients with valvular disease can be treated with a wider range of surgical options. The advent of transcatheter access offers new hope for patients with high surgical risk, severe tricuspid regurgitation, severe right ventricular dysfunction, or severe pulmonary hypertension. Transcatheter surgery has been equally successful in the treatment of patients with tricuspid regurgitation [105, 106, 107, 108, 109]. Also for patients who have failed treatment for tricuspid regurgitation, percutaneous intervention gives them a chance to remedy the situation [110, 111, 112]. Reoperation should not only take into account the patient’s previous surgical approach, but also pay special attention to the structural characteristics of the tricuspid valve. As this section is outside the scope of this article, it will not be discussed in detail. There are numerous percutaneous tricuspid valve surgical options, and we have listed some of the products that are currently widely used in clinical practice (**Supplementary Fig. 1**).

## 7. Conclusions

Surgery is an effective treatment for FTR and can effectively improve the degree of TR and prognosis of TR patients. Tricuspid annuloplasty is favored by more cardiovascular surgeons in clinical practice because of its better resulting plasticity and long-term results. However, these surgical procedures do not completely cure TR and do not guarantee that it will not recur after surgery. Transcatheter tricuspid valve therapy could play a clinical role as a complement to surgical procedures.

## Abbreviations

FTR, functional tricuspid regurgitation; TR, tricuspid regurgitation; TV, tricuspid valve; RV, right ventricle; LV, left ventricle.

## Supplementary Material

Supplementary material associated with this article can be found, in the online version, at https://doi.org/10.31083/j.rcm2505182.
