# Supplementary figures and images for: Surgical Treatment Strategy of Functional Tricuspid Regurgitation
Source: Rev Cardiovasc Med. 2024 May 21;25(5):182. doi: 10.31083/j.rcm2505182 (PMC11267193; doi:10.31083/j.rcm2505182)

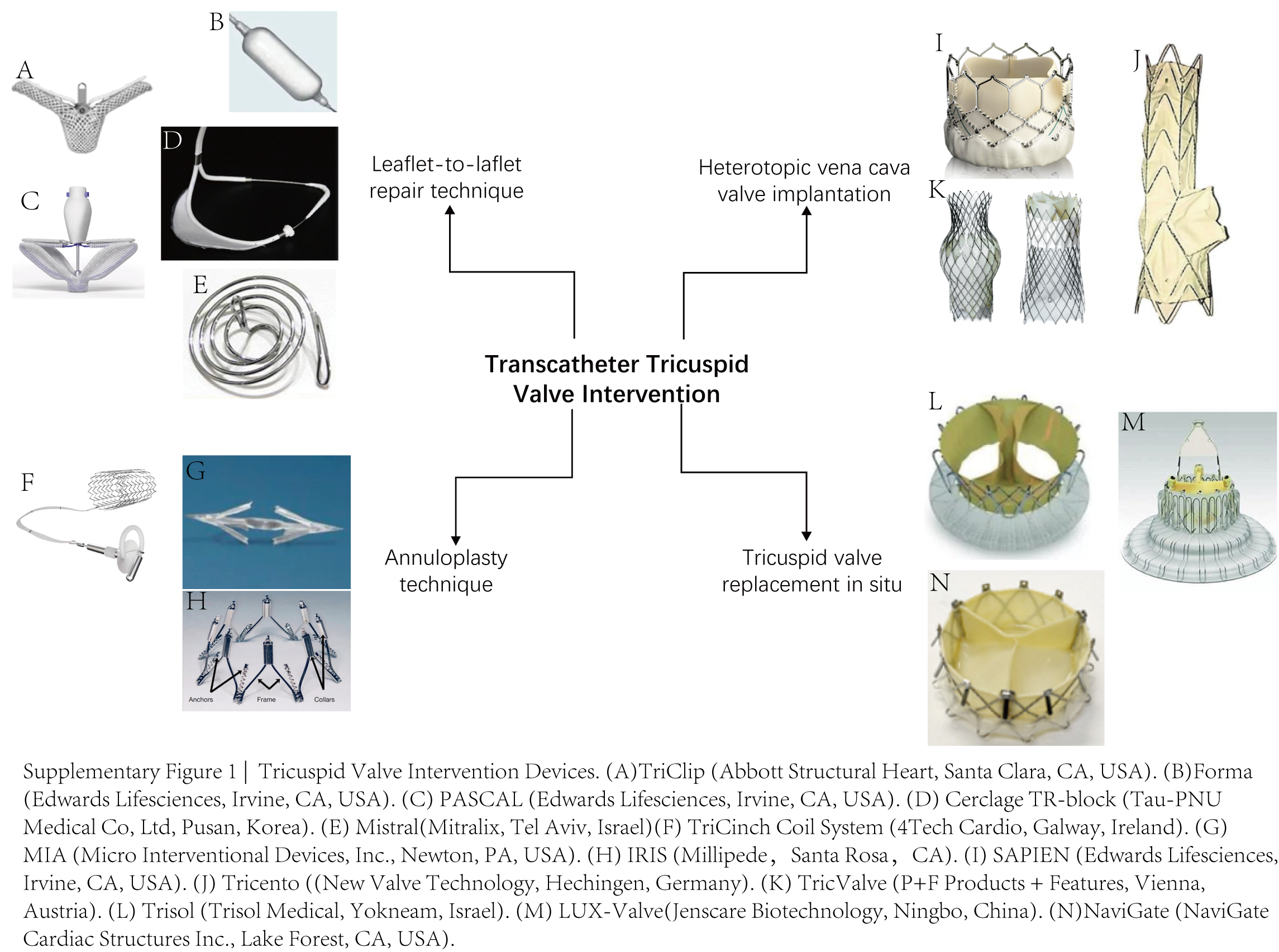

Supplement: Supplementary file 1 [file 2153-8174-25-5-182-s1.zip › 2153-8174-25-5-182-s1/Supplementary Material.tif]
